# The Implications of Motor and Cognitive Inhibition for Hot and Cool Executive Functions: The Case of Quadrato Motor Training

**DOI:** 10.3389/fpsyg.2020.00940

**Published:** 2020-05-19

**Authors:** Rotem Leshem, Antonio De Fano, Tal Dotan Ben-Soussan

**Affiliations:** ^1^Department of Criminology, Bar Ilan University, Ramat Gan, Israel; ^2^Research Institute for Neuroscience, Education and Didactics, Patrizio Paoletti Foundation, Assisi, Italy

**Keywords:** motor inhibition, quadrato motor training, executive functions, self regulation, sensorimotor training

## Abstract

Enabling the ceasing of ongoing or prepotent responses and the controlling of interference, motor inhibition facilitates the development of executive functions (EFs) such as thought before action, decision-making, self-regulation of affect, motivation, and arousal. In the current paper, a characterization is offered of the relationship between motor inhibition and the executive functioning system, in the context of a proposed division into predominantly affective (hot) and cognitive (cool) components corresponding to neural trajectories originating in the prefrontal cortex. This division is central to understanding the effects of a specifically-structured sensorimotor movement training practice, known as Quadrato Motor Training (QMT), on hot and cool EFs. QMT’s effects on crucial mechanisms of integrating different EF components are discussed.

## Introduction

Motor and cognitive development’s close relationship (Sibley and Etnier, 2003; Pesce et al., 2016; Stein et al., 2017) is exemplified by the role of motor inhibition in the development of executive functions (EFs) (Hammond et al., 2012). As high-order cognitive functions, EFs (e.g., working memory, inhibition, planning, active monitoring, set shifting; Miyake et al., 2000; Diamond, 2013) contribute to goal-directed behavior while helping limit impulsive responses and regulate emotions (Riggs et al., 2013; Blair, 2016; Leshem, 2016; Leshem and Yefet, 2019). By inhibiting ongoing or prepotent responses and controlling attentional interference (Bickel et al., 2012; Bari and Robbins, 2013; Leshem and Yefet, 2019), motor inhibition facilitates EF development: thought before action, decision-making, and self-regulation of affect, motivation, and arousal (Barkley, 1997).

Therefore, motor inhibition, developed through experiences that involve body movements, is supposed to integrate motor and cognitive development. Motor experiences elicit different structural and functional changes, including physiological changes, such as enhanced cerebral blood flow (Stein et al., 2017) and changes in neurotransmitter release (Goldstein, 2006; Miranda, 2007; Winter et al., 2007). The interrelationship between motor and cognitive functions is further reflected by their simultaneous neuronal activation during complex or novel tasks requiring fast reactions or changing conditions (Diamond, 2000; Koziol et al., 2014; Leisman et al., 2016; Stein et al., 2017).

This mini-review characterizes the relationship between motor inhibition and EFs in the context of a proposed division into predominantly affective and cognitive components. This division is central to understanding the effects of structured movement practices on EF-based abilities.

## Functionality- and Cognitive-Based Divisions in Executive Functioning

EFs are traditionally considered purely cognitive top-down processes. They are typically categorized into either abstract cognitive functions (e.g., general attention abilities, such as switching attention; Heaton et al., 1993; Kenemans et al., 2005) or those that operate in highly motivated or emotionally salient contexts (Zelazo and Müller, 2002). Accordingly, a distinction has been proposed between “cool EFs,” elicited by abstract, decontextualized tasks lacking significant affective or motivational components (Zelazo and Carlson, 2012; Tsermentseli and Poland, 2016), and “hot EFs” (Zelazo and Müller, 2002), involved in emotions, beliefs, or desires, such as those associated with reward and punishment, social behavior, and emotional components of decision-making (Leshem, 2016).

Neuroscientific studies suggest a distinction between cool and hot EFs, reflected in how they are traditionally measured (Bechara et al., 2000; Berlin et al., 2004; Anderson, 2010; De Luca and Leventer, 2010; Zelazo and Carlson, 2012). Patients with specific brain damage (e.g., orbitofrontal cortex) may show intact cool EFs (assessed by classical tests such as the WCST) but impaired hot EFs (assessed by tests like “the Iowa gambling task” (e.g., Bechara et al., 1994) or vice versa (Zelazo and Carlson, 2012).

This functional distinction may reflect a structural one, as neural systems underlying EFs appear to vary as a function of motivation. It is generally accepted that EFs are largely governed by the pre-frontal cortex (PFC) and its reciprocal interactions with other cortical and subcortical brain regions (Diamond, 2000; Miyake et al., 2000; Blair, 2016). Accordingly, hot and cool EFs are theorized to be associated with differential neural trajectories originating in ventral-medial and dorsolateral PFC sections, respectively (Zelazo and Müller, 2002).

Hot EFs are generally associated with the paralimbic cortex, comprised of the ventromedial PFC (VMPFC) and caudal orbitofrontal cortex (OFC). Closely connected to limbic structures (e.g., amygdala, hypothalamus) these areas provide descending input to midbrain structures (Barbas, 2000) and are involved in inhibition, emotion, and reward processing − suggesting a behavioral self-regulation role. Numerous case studies illustrating the effects of damage to these areas support this notion (Stuss and Levine, 2002; Fellows, 2004; Bechara, 2005).

In contrast, cool EFs are thought to recruit the lateral PFC, including the dorsolateral PFC (DLPFC), involved in attentional control, planning, working memory, spatial and conceptual reasoning, and learning (Stuss and Levine, 2002; Fellows, 2004; Banfield et al., 2004; Toplak et al., 2005; Crews and Boettiger, 2009).

Moreover, distinct EF components appear to demonstrate different developmental trajectories. Age-related improvements seem to emerge later and more gradually for hot EFs than cold, suggesting varied development courses. EFs are known to develop both linearly and nonlinearly during childhood and early/late adulthood, associated with underlying neural development and maturation (Best and Miller, 2010; Taylor et al., 2015). Progressively less recruitment of PFC regions is normal with increasing age (Somerville et al., 2011), presumably due to more diffuse neural engagement and less specialization at younger ages (Luna et al., 2013; Casey, 2015). As the brain continually reorganizes, mainly during adolescence, necessary neural connections are strengthened and unnecessary ones are pruned, creating more efficient focal recruitment of PFC regions (Shulman et al., 2016). According to the orthogenetic principle (Werner, 1978), executive functioning may be an initially unified construct (Wiebe et al., 2008; Hughes et al., 2009; Wiebe et al., 2011) that differentiates and specializes over time (Miyake et al., 2000; Johnson, 2011; Howard et al., 2015), producing distinct hot and cool EFs.

Deficits in hot and cool EFs may engender different psychopathologies and developmental outcomes (Anderson, 2002; Sonuga-Barke, 2005). When brain reorganization processes do not progress normally, diffuse engagement of neural regions may continue, resulting in insufficiently differentiated EFs (Johnson, 2011; Zelazo and Carlson, 2012). Likewise, if neural circuitry is damaged due to extrinsic injury, resulting behaviors can reflect earlier stages of EF development; such clinical expressions generally include disruption in both hot and cool EFs, such as deficits in delay gratification, inability to anticipate consequences, and verbal and behavioral disinhibition (Zelazo and Carlson, 2012).

## Integration Between Hot and Cool EFs in Adaptive Functioning

While distinct, hot and cool EFs also appear interdependent, working together in a coordinated system (Zelazo and Carlson, 2012). Dysfunction in both hot and cool EFs is apparent in a broad range of neurodevelopmental and psychopathological conditions (Snyder et al., 2015; Malloy-Diniz et al., 2017) such as autism (Gilotty et al., 2002; Luna et al., 2007; Johnston et al., 2019), ADHD (Swanson, 2003; Tsal et al., 2005; Barkley, 2010; Stern et al., 2017), PTSD (Aupperle et al., 2012; Olff et al., 2014) and anti-social personality disorder (Morgan and Lilienfeld, 2000; Ogilvie et al., 2011).

Known to affect systems associated with either hot or cool EFs at the neural level, these disorders nevertheless appear to manifest both components at the clinical and behavioral level, suggesting interdependency. For example, while cool EFs are a focus of autism spectrum disorder research (Kouklari et al., 2019), a crucial role for hot EFs has also been proposed (Kouklari et al., 2019). Zelazo and Müller (2002) theorize that autism is characterized by primary deficits in hot EFs with secondary impairments in cool EFs. Conversely, anti-social personality disorder and/or psychopathic traits are generally associated with hot EF deficits, such as response reversal, sensitivity to reward, and affective decision-making (Mitchell et al., 2002; Blair, 2010; Carré et al., 2013) despite mixed findings in both EF deficits among individuals exhibiting anti-social behavior (see De Brito et al., 2013; Delfin et al., 2018).

Interdependency between hot and cool EFs suggests that impairments in one EF type may lead to impairments in the other. Therefore, integration and balance between the two EF components and their underlying neural systems may be essential to adaptive functioning and well-being. A specific form of structured sensorimotor training may play a significant role in achieving this integration.

## Enhancing Executive Functioning Through Motor Inhibition: Quadrato Motor Training and the Ability to Wait

Quadrato Motor Training (QMT) is a non-aerobic, coordination-demanding, and cognitively-engaging movement practice developed by Patrizio Paoletti (Ben-Soussan et al., 2015b). Practitioners are required to alternate between dynamic movements and static postures, while focusing attention and awareness on their bodies in the present moment and excluding all the other thoughts. QMT is conducted on a 50 × 50 cm square known as the Quadrato space. Its corners are labeled with the numbers 1–4. Practitioners are required to either produce or inhibit a motor response in the Quadrato space, based on specific verbal instructions presented in an audio recording. The motor responses are steps in one of three possible directions: right or left; forward or backward; or diagonally. For example, a verbal instruction can be “1–2,” which directs the practitioner to take a step forward from corner number 1 to corner number 2. When the two numbers of the verbal instruction are the same (e.g., “1–1”), the practitioner must inhibit the impulse to move upon hearing the voice command and wait for the next instruction. The inhibitory control (cognitive and motor) required to make a decision based on cognitively processed information related to the specific verbal instruction is one of the main features of the QMT. Inhibitory control is also involved in continuing to the next instruction rather than stopping when a mistake occurs (De Fano et al., 2019).

Diamond and Ling (2016) claimed that practices aiming to improve cognition in general, and EFs in particular, must not only recruit cognitive resources, but challenge them continually. It therefore stands to reason that QMT, which is associated with continual cognitive challenges, would improve cool EFs. Indeed, this was demonstrated in previous research using abstract, decontextualized problem-solving tasks absent significant affective or motivational components, such as the Alternate Uses Test (AUT; Guilford, 1967; Ben-Soussan et al., 2013, 2015a; Venditti et al., 2015; Piervincenzi et al., 2017). Both single session and protracted QMT practice have produced increased ideational flexibility (Ben-Soussan et al., 2013, 2015a; Venditti et al., 2015) − the ability to produce creative ideas by shifting from one meaningful category to another (Diamond, 2013). A prominent measurable dimension of divergent thinking (Guilford, 1967), this ability to change perspectives relies on inhibitory control; in order to effectively “think outside the box,” other perspectives previously loaded in working memory must be inhibited (Diamond, 2013).

QMT-induced gains in ideational flexibility likely result from motor and cognitive inhibition acquired through the cognitively-engaging motoric practice. Indeed, interventions involving only the cognitive component of QMT practice (participants responding verbally to QMT instructions rather than with movement) did not significantly change ideational flexibility (Ben-Soussan et al., 2013, 2015a). Conversely, practicing a motor component similar to the QMT (i.e., taking steps) but without cognitive effort (i.e., reduced reaction choices) also did not change ideational flexibility (Ben-Soussan et al., 2013, 2015a; Venditti et al., 2015). This suggests that neither motor experience, nor cognitive challenge, alone is sufficient to enhance cool EFs. Benefits appear to result from the combination, as evidenced by physical activity research indicating that more effective improvement in executive functioning resulted from combined cognitive, physical, and emotional engagement, than on cognitive stimulation or physical activity alone (Pesce, 2012; Tomporowski and Pesce, 2019).

Moreover, cognitive benefits of QMT are not limited to cool EFs. QMT also enhances self-efficacy (Paoletti et al., 2017; Piervincenzi et al., 2017) and affect balance (Paoletti et al., 2017), which are both closely related to higher-order cognitive functions and self-regulation (Schunk and Zimmerman, 2007; Kessler and Staudinger, 2009).

Research shows that one week of intense QMT combined with a breathing meditation enhances general self-efficacy, compared to the breathing meditation alone (Paoletti et al., 2017). Moreover, the combination of QMT and breathing meditation improved affect balance, shifting it toward more positive emotions (Paoletti et al., 2017). These results support the notion that cognitively-engaging movement-based experiences can improve hot, as well as cool, EFs.

The impact of motor-cognitive-affective activity elicited by QMT was explored using semi-structured oral interviews (Ben-Soussan et al., 2017). Following QMT practice, participants report increased experiences of attention, mindfulness, ability to wait, positive emotions, and bodily harmony. They also report experiences of spontaneous visualization, intuition, and sense of wonder, which have been categorized as altered states of consciousness and are similar to experiences commonly elicited during meditation practices (Wallace, 1999). This further supports QMT’s potential enhancement of hot EFs. QMT may be considered an embodied cognitive training and “mindful movement” (Ben-Soussan et al., 2014; De Fano et al., 2019). Like other mindfulness-based practices, mindful movement is mainly characterized by a focus on movement in the present moment while excluding other thoughts, inclusion of some form of body movement, and focus on breathing (De Fano et al., 2019); nevertheless, the existing studies related to QMT of which we are aware did not provide direct, explicit instructions related to these components. Yet, each participant clearly had to be mindful of the location of their body in the Quadrato space and, therefore, we cannot exclude the importance of body awareness and interoception, which, in turn, can affect hot EFs. Russell and Arcuri (2015) suggest mindful movement practices involve key aspects of mindfulness such as preparation and execution of movement, regulation of attention, working memory, and their relationship to mind-wandering, an opposite construct to mindfulness (Mrazek et al., 2012). QMT involves each of these aspects: regulation of divided attention, working memory updating (e.g., noting one’s current location to know where to move to next), and prevention of mind-wandering via a need to be “in the here and now” due to constantly updating commands (Ben-Soussan et al., 2014; De Fano et al., 2019). Mindful movement training engages “higher-order” inhibition and response selection that underlie attention and cognitive control) that requires moment-by-moment sensorimotor updating (Clark et al., 2015; Kimmel and Rogler, 2018). In line with this, QMT requires second-by-second mindful awareness for attending the upcoming next command (Ben-Soussan et al., 2014; De Fano et al., 2019).

## QMT as a Means of Integrating Cool and Hot EFs

QMT’s potential benefits for both cool and hot EFs are further supported by research on functional and structural brain changes. As noted above, cool and hot executive processes are, respectively, associated with specific areas of the PFC. Those areas are interconnected with numerous regions throughout the brain, associated with changes in behavior and cognition that fall within the broader area of EFs (Barbas and Zikopoulos, 2007). Goal-directed, purposeful EFs support cognitive and affective states with purported association with differential neural trajectories originating in dorsolateral, and ventral and medial, PFC sections, respectively (Zelazo and Müller, 2002; Blair, 2016).

Accurate execution of QMT involves concurrent motor activation and response inhibition. In turn, response inhibition is mediated both by the cool EF system responsible for planning, control, and execution of voluntary movements, and the hot EF system associated with processing and regulating behavior involving emotional content (Bush et al., 2000; Etkin et al., 2011). Executing specifically structured movements can lead to integrated communication between brain areas associated with cognition and emotion. Accordingly, QMT generates changes in neural activities, such as frontal alpha activity and contingent negative variation amplitude (Ben-Soussan et al., 2013; Lasaponara et al., 2019), known to be closely related to planning, decision making, and moral judgment (Harung et al., 2009; Travis et al., 2011), all of which require both cool and hot EFs; thus supporting the hypothesis that QMT can improve cool and hot EFs.

Ben-Soussan et al. (2013) showed that improvements in ideational flexibility were concurrent with enhanced intra- and inter-hemispheric connectivity, expressed in increased synchronization between brain regions located in the same or different hemispheres, respectively. Increased neural synchronicity was specifically related to bilateral fronto-temporal networks and frontal areas in the alpha band (8–12 Hz), and was confirmed in a later study by the same group (Lasaponara et al., 2017). Accordingly, QMT may positively affect cool EFs by increasing frontal alpha connectivity. One should keep in mind that so far this line of studies examined changes in baseline EEG activity rather than functional EEG activity during a behavioral task. Future studies should examine this also during task conditions.

These findings align with research showing enhanced attention induced by an attentional training program and mediated by increased functional connectivity in frontal regions (Liu et al., 2019). Similarly, Basharpoor et al. (2019) found a positive association between high executive functioning, including inhibitory control, and increased alpha activity in frontal regions of right and left hemispheres. Increased functional synchronicity in the alpha band was found not only in relation to frontal areas; following QMT, the limbic network was also found more synchronized with both hemispheres’ frontal areas, compared to pre-training (Lasaponara et al., 2017).

As noted above, the limbic system is involved in emotional experience, motivation, learning, and memory formation (Isaacson, 1982; LeDoux, 2000). In this regard, there is a line of research within the theories of consciousness and high-order cognition that argue that emotions are not built-in but are higher-order states instantiated in cortical circuits. They are not triggered but created. Because the limbic cortices are strongly interconnected and position in hierarchical cortical information flow, they can contribute to the neural basis of conscious access and may be a source of emotional experience and influence the coordination and regulation of cognitive-emotional processes (Chanes and Barrett, 2016; Kovner et al., 2019; for extensive reading see Barrett, 2017; LeDoux and Brown, 2017). The QMT-associated increase in limbic-frontal functional connectivity may reflect its effectiveness in integrating the “coolest” of cognitive functions with the “hottest.” Possibly promoting adaptive behavioral responses, this could underlie the QMT-induced increase in general self-efficacy and affective balance (Paoletti et al., 2017; Piervincenzi et al., 2017).

Notably, QMT has been found to increase white matter integrity in several tracts [e.g., superior longitudinal fasciculi (SLF) and uncinate fasciculi (UF); Piervincenzi et al., 2017] that connect the PFC with the medial temporal lobe (Kovner et al., 2019), which is associated with numerous disorders of maladaptive behavior and low emotional control, including obsessive-compulsive disorder and PTSD (Jenkins et al., 2016). Further, enhanced SLF white matter integrity was positively associated with improvements in ideational flexibility (Ben-Soussan et al., 2015a) and general self-efficacy (Piervincenzi et al., 2017). QMT-induced improvement in UF white matter integrity was also positively correlated with first-person reports of experiencing reduced mind-wandering (Ben-Soussan, 2019). These findings support the possibility that QMT facilitates integration between cool and hot EFs. Future studies should examine whether QMT-induced enhanced ideational flexibility and self-efficacy, as well as reduced mind-wandering, in healthy adults could potentially ameliorate maladaptive pathologies such as in PTSD or OCD.

## Concluding Remarks

This mini-review presented a proposed division for predominantly cool and hot EFs and introduced QMT, a sensorimotor training paradigm, as a means of enhancing healthy development (Levit-Binnun et al., 2013) by integrating these EF components.

QMT’s requirement of smoothly executed goal-directed behaviors in response to predetermined verbal instructions separated by interstimulus intervals (ISIs), which are known to increase the duration of attention (Leckart et al., 1970), differentiates it from other mindful movement practices (Ben-Soussan et al., 2019).

QMT requires second-by-second response inhibition (Ben-Soussan et al., 2014; De Fano et al., 2019), and thus requires high order cognitive function, which is more related to cool EFs. Notwithstanding, evidence suggests training that requires attentional abilities also leads to enhancement of hot EFs. As hot EFs may benefit from physical activity, especially when executed in emotionally evocative settings, future research is encouraged to uncover the effects of physical activity on a broader range of EFs.

## Author Contributions

TB-S and RL conceptualized the manuscript. TB-S, RL, and AD wrote the manuscript.

## Conflict of Interest

The authors declare that the research was conducted in the absence of any commercial or financial relationships that could be construed as a potential conflict of interest.
